# Clustered Intrinsic Connections: Not a Single System

**DOI:** 10.3389/fnsys.2022.910845

**Published:** 2022-06-03

**Authors:** Kathleen S. Rockland

**Affiliations:** Department of Anatomy and Neurobiology, Boston University School of Medicine, Boston, MA, United States

**Keywords:** axon collaterals, cortical column, diversity, horizontal connections, like-to-like, pyramidal cells, recurrent

## Introduction

In both sensory and non-sensory cortical areas, pyramidal neurons typically have horizontal collaterals spatially extending in a 1.0–3.0 mm radius from the cell body; and in primates and cats, these often have a patchy or discontinuous conformation (aka “daisy,” Douglas and Martin, 2004). In the primary sensory cortices, where these have been most extensively investigated, the discontinuous topology is evocative of functional domains (Angelucci et al., 2002; Douglas and Martin, 2004; Angelucchi et al., 2017). The underlying anatomy, however, is complex, as best visualized at the level of individual axons. The fact that patchy intrinsic connections occur in frontal, parietal, and temporal areas might imply a wider functional significance; and the frequently remarked axonal heterogeneity within visual areas is also suggestive of functional diversity (Buzas et al., 2006: “…the spatial and orientation-specific intrinsic excitatory network is composed of neurons following various connectivity rules;” “Thus, instead of treating the lateral connections as a single homogeneous network, the real clue to its structure and function may lie in its heterogeneity of connections,” Martin et al., 2014; and discussion in Kisvarday, 2016; Chavane et al., 2022).

## Background

Horizontal clustered connections have been investigated by two complementary techniques. One is from bidirectional extracellular tracer injections, which provide averaged data at the populational level (see Buzas et al., 2006 for further discussion). The resulting patches each consist of anterogradely labeled terminations from the injected locus intermingled with retrogradely filled neurons that project reciprocally back to that locus, and possibly as well (but to be determined) to the co-labeled patches (e.g., Rockland and Lund, 1983; Lund et al., 1993; Kritzer and Goldman-Rakic, 1995; Pucak et al., 1996; Tanigawa et al., 2005). The patches are not uniform, but vary in size, shape, and number of labeled neurons and terminations, such that there is a progressive diminution of both with distance from the injection site.

The shape and size of the clusters at the populational level is most clear in histological sections tangential to the cortical surface and is area-specific. In macaques, patches are overall smaller and less spatially extended from an injection site in V1 than in TEO or TE (Tanigawa et al., 2005; Wang et al., 2017). In V2 and V4, the populational layout appears more “reticular,” consisting of discrete patches with short stripelike arrays (Rockland, 1985; Yoshioka et al., 1992). In prefrontal cortex (PFC), the global pattern is more stripelike (Levitt et al., 1993; Lund et al., 1993; Pucak et al., 1996; Melchitzky et al., 1998).

The developmental timecourse of the characteristic clustering is species and area variable (reviewed in Wang et al., 2017; Danka Mohammed and Khalil, 2020); but significantly, horizontal collaterals are overall discernible relatively early in development. This argues for an initiating nfluence of spontaneous activity and/or molecular factors, with a later shaping by visual features such as ocularity or orientation specificity (Luhmann et al., 1986; Callaway and Katz, 1990; Durack and Katz, 1996; Schmidt et al., 1999; Katz and Crowley, 2002). In the visual cortex of ferrets, crude clusters are apparent at postnatal day (PD) 34, prior to eye opening (at PD30-32); and better defined adult-like clusters can be identified at PD45 (Durack and Katz, 1996). In cats, crude clusters are detectable at PD8, appearing adult-like at PD36-38 (Callaway and Katz, 1990). In areas V1 and V2 of macaques, patchy horizontal axons can be discerned around embryonic day 140–145, 3 weeks before birth (Coogan and Van Essen, 1996). In human V2, long-range connections in the superficial layers are present prenatally at 37 weeks gestation, and denser and more adult-like by PD9 (Burkhalter, 1993). In human V1, however, short collaterals in layers 2/3 emerge later, after 16 weeks postnatal, reaching adult-like patchiness sometime before 15 months of age (Burkhalter, 1993; Burkhalter et al., 1993).

The second techique, the focus of the present Opinion, is intracellular injections, where the full axonal (or, at least, full intrinsic) arborization of a single neuron is visualized. This gives primarily an anterograde perspective. A consistent finding with this level of resolution is that “the distal clusters vary in number from cell to cell and for an individual cell, no two clusters are the same size or contain the same number of boutons” (Buzas et al., 2006; Martin et al., 2017). In consequence, individual neurons differentially and presumably selectively contact postsynaptic neurons that are not only spatially dispersed but possibly functionally disparate. What appears to be a conspicuous modularity at the populational level, at the higher resolution of single axons actually presents as a prominent heterogeneity and complex webwork of convergence and divergence.

Whole cell visualization of both intrinsic and extrinsic collateralization is now possible in rodents (Kita and Kita, 2012; Winnubst et al., 2019), but not yet for NHP. That some pyramidal neurons have only intrinsic collaterals has been consistently reported (Yabuta and Callaway, 1998; Martin et al., 2014), but more detailed information is lacking as to comparison and co-variability of intrinsic collaterals with different extrinsic connections (Rockland, 2020; Vanni et al., 2020; and for callosal connections, see Schmidt, 2016).

## Supragranular Neurons

For supragranular pyramidal neurons, multiple studies report a mixed axonal architecture of a bouton-dense local or home cluster, spatially coincident with the soma of the individual neuron, and a variable number of distal terminal clusters (cat V1: 4-8, Kisvarday and Eysel, 1992; up to seven; Binzegger et al., 2007; macaque TE, up to 43, Tanigawa et al., 2005). These are spatially extended from the soma for ~1.0 mm in cat V1, and up to 8.0 mm in macaque area TE (Tanigawa et al., 2005; Wang et al., 2017). The home cluster is larger and has the largest number of terminations, being about 70% of the total number for a given neuron (cat visual cortex: Buzas et al., 2006; Binzegger et al., 2007; Martin et al., 2017). In area V1 (and percentage unknown for other areas), about 20% of filled pyramidal neurons, possibly comprising a distinct subtype, only have a local, home cluster (macaque: Yabuta and Callaway, 1998; cat: Martin et al., 2014).

The mix of home and distal clusters might be significant in the context of feature responsivity (“like-to-like” connectivity); for example, a like-to-like connectivity bias at short distances from a parent soma (related to greater synaptic convergence with neighbor neurons?), but like-to-all at greater distances (Chavane et al., 2022). The neuron-specific organization of intra-columnar functionally redundant or synergistic interactions remains under active investigation (e.g., Nigam et al., 2019). A single-cell perturbation study in mouse V1 reports that presynaptic neurons suppressed activity more when postsynaptic neighboring neurons were similarly tuned than for dissimilarly tuned neurons. A “like-suppresses-like” motif was suggested as reducing redundancy in population activity (Binzegger et al., 2007; and Chettih and Harvey, 2019: “a spoke-like network architecture…reducing signal correlation and redundancy”).

**Figure 1:**
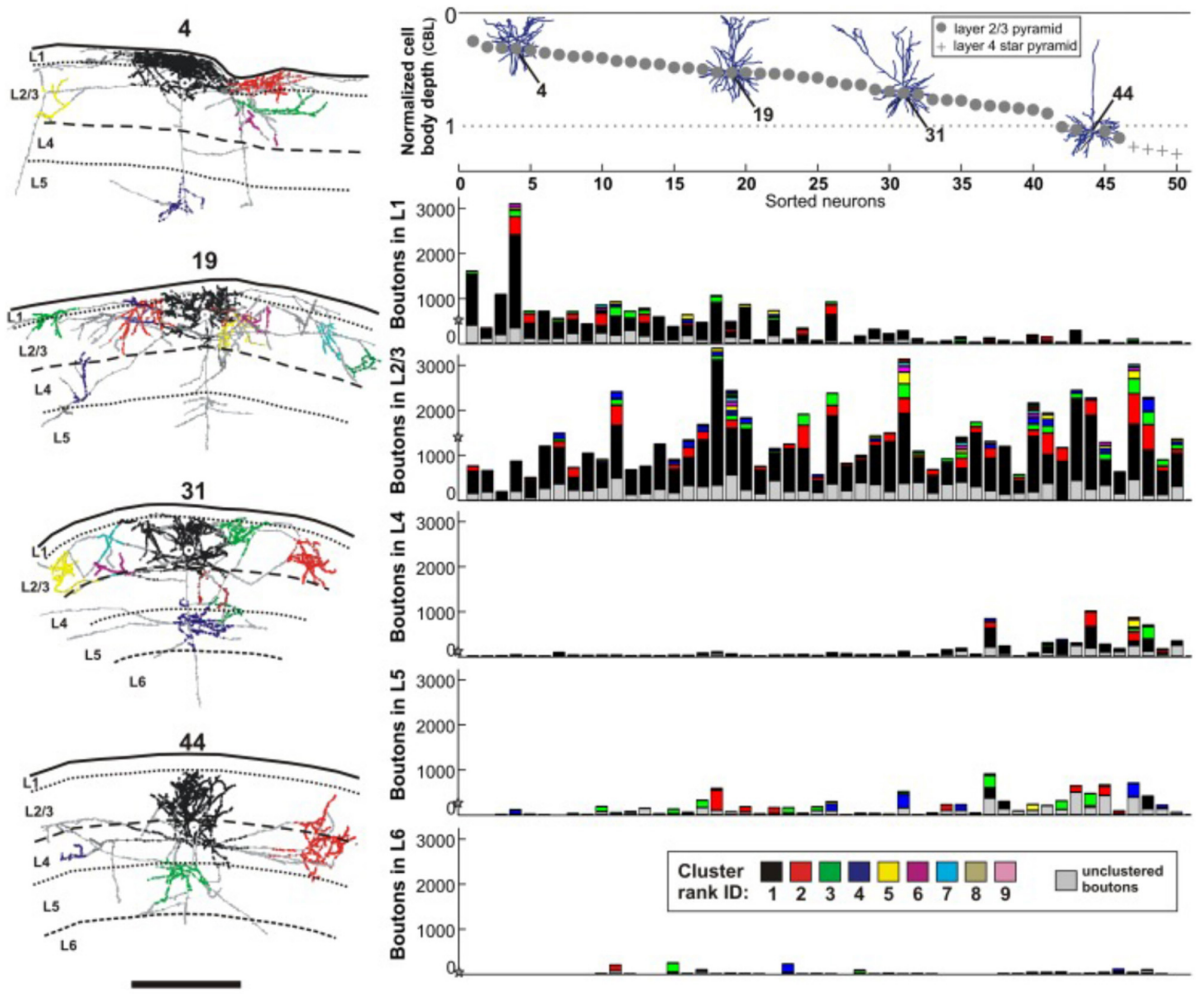
Reconstructions, at left, of four intracellularly filled supragranular neurons in cat V1 (neuron 4, 19, 31, 44), at variable depths from the pia surface. Laminar distribution and spatial spread of boutons are non-stereotyped. Views are for coronal sections, with the home clusters in black. Schematic at upper right depicts the same four neurons in relation to depth from the pia surface, where each neuron is given a specific identity along the x-axis. Below, five histograms for each of five cortical layers (L1, L2/3, L4, L5, L6) where the number of encountered boutons per layer is plotted along the y-axis. The mean number of boutons per layer is depicted with a star on the y-axis. Scale bar = 1.0 mm. From Martin et al., 2017 (their figure 3).

The sublaminar distribution of terminations for any one neuron is reported to vary in correlation with the soma depth from the pia (Figure 1; and Martin et al., 2017). Neurons close to the pia have a larger number of boutons in layers 1 and 2; but, in a gradient-wise fashion, neurons progressively deeper in layer 3 have the majority of their boutons commensurately shifted in depth. While the postsynaptic targets are likely to be other supragranular pyramidal neurons irregardless of the depth of the presynaptic neuron, there is evidentally depth-wise heterogeneity of postsynaptic dendritic locations such that distal apical tufts may be targeted by superficially placed neurons, but not by those more deeply situated.

Distal clusters commonly receive uo to 10 converging branches from the same neuron, which contribute differing proportions of boutons to a single cluster (cat visual cortex: *n* = 10 neurons, Martin et al., 2014; *n* = 33 neurons; Martin et al., 2017, *n* = 50 neurons). Intracellular fills reveal that a single branch of the same neuron can diverge to more than one cluster, thereby contacting spatially separated postsynaptic neurons (cat visual cortex: Kisvarday and Eysel, 1992). The distal patches of one neuron will have membership in other local networks. Each “distal” patch for a given neuron is within the “home cluster” for other neurons. The image is of an intricate system of converging/diverging connections, anchorable to a designated pyramidal neuron but interweaving with multiple spatially offset networks.

### Branch Specificity

There are further sharp topological differences within the intrinsic axon arborization of an individual neuron. The same supragranular neuron can have linear collaterals with boutons scattered along the length, together with collaterals having a more circumscribed terminal cluster (Buzas et al., 2006; Koestinger et al., 2017). The linear collaterals sparsely contact a row of neurons, seemingly without regard to any columnar organization, whereas the clustered terminations can be assumed to make a higher number of contacts with a more spatially delimited set of postsynaptic neurons. The architectural significance of this mixed linear-and-clustered geometry is unclear; but, intriguingly in the context of cortical organization, something similar has been observed for some feedback axons (e.g., V4 to V1, figure 12 in Rockland et al., 1994) and for some pulvinocortical axons in V2 (figures 8, 17 in Rockland et al., 1999).

In cat V1, the intrinsic collaterals of a single neuron can be myelinated or unmyelinated in their trajectory to distal clusters (Kisvarday and Eysel, 1992; Koestinger et al., 2017). Myelination has commonly been investigated in the context of conduction velocity, but is increasingly considered with other processes such as adaptive remodeling in response to different conditions of sensory and non-sensory processing (Fields, 2015; Bonetto et al., 2020; Yang et al., 2020; de Faria et al., 2021). A high resolution study of parvalbumin+ basket cells, using immunofluorescent array tomography, reported complex features of myelination of local collaterals and, more particularly, a complex meshwork of multiple axonal paths taken by the axon of a single inhibitory neuron to a single postsynaptic neuron (Micheva et al., 2021). The multiplicity of axon paths was viewed as possibly subserving a temporally staggered arrival of input from the same parent neuron. A similar mechanism might be proposed for pyramidal neurons with more or less tortuous trajectories of myelinated and unmyelinated collaterals (Martin et al., 2017), although in this case, spatially separate postsynaptic neurons are targeted.

## Infragranular Neurons

Single cell data for infragranular neurons are comparatively sparse; but the evidence suggests that (1) the organization of their intrinsic collaterals is more diverse than that for supragranular neurons, (2) that these may be less clustered, and (3) the parameters may be strongly correlated with the connectional identity of the parent neuron. In layer 5 (cat auditory cortex: Ojima et al., 1992; cat visual cortex: figure 4 in Buzas et al., 2006) likely corticothalamic neurons (type 2) in the upper part of layer 5 were reported to have spatially extended distal collaterals but few proximal terminations. This perisomatic termination-sparse gap is in striking contrast to the termination-dense home column more typical of supragranular neurons, and presumably influences the spatial configuration of the postsynaptic neuron set. No examples have been illustrated so far of perisomatic termination-sparse gaps for supragranular pyramidal neurons. Likely corticostriatal neurons in layer 5 had both extended collaterals and dense terminations in the home cluster, as was also reported for a subpopulation of neurons in the deeper part of layer 5 that lacked extrinsic connections (Ojima et al., 1992).

In layer 6, intrinsic collateral distribution was reported as more variable, with likely corticothalamic neurons (type 1) having proximal recurrent collaterals only (Ojima et al., 1992 and see Karube et al., 2017: three types of layer 6 neurons in cat area 18). As discussed elsewhere (Rockland, 2019; and, in a different system, see Parent et al., 2000), at least for the two distinct types of cortiocothalamic neurons, there seems to be an inverse spatial ratio of intrinsic and extrinsic collaterals (for type 2: extended intrinsic and delimited extrinsic, but the reverse for type 1). Large infragranular Meynert cells in macaque V1, some of which project to the pulvinar and/or superior colliculus also have unusually extended intrinsic collaterals, without a conspicuous home cluster (figure 12 in Rockland and Knutson, 2001; Liu et al., 2014).

Supragranular neurons commonly have collaterals in the deeper layers, and collaterals from some infragranular neurons ascend to the supragranular layers (Gilbert and Wiesel, 1983; McGuire et al., 1991; Ojima et al., 1991, 1992). These interlaminar terminations are numerically fewer and more spatially restricted than those in the home layer of an individual neuron, and often are not patchy. Thus, a putative columnar volume has laminar-specific substructure. This applies to other connections with bilaminar terminations. A subset of geniculocortical axons have a smaller collateral in layer 6, subjacent to the main arbor in layer 4 (Blasdel and Lund, 1983). Axons projecting from V1 to MT have arbors in layer 6 which are consistently offset from terminations in layer 4 and, again, smaller (Rockland, 1989).

## Substructure of a Cortical Column

Only limited anatomical data are available concerning the fine organization of a putative cortical columnar network; there is no “brainbow” mapping of multiple individually identified axon collaterals. The complex spatial arbors and convergent-divergent architecture suggests a fingerprint-distinctive substructure, where “column-specific” proportions of synapses derive from the individual, identified neurons. Buzas et al. (2006) similarly noted that only a subset of potential postsynaptic loci are reached by the same population of intrinsically projecting neurons; i.e., something of a combinatoric architecture, where intrinsically terminating arbors in the same cortical column may only partially overlap (i.e., converge and diverge to a wider set of “columns”).

A fine dissection has been published of a putative “column” of extrinsic terminations in a delimited focus in parietal cortex (Rockland, 2002). A single neuron in a temporal cortical injection site terminates in two spatially separate foci. The larger focus is formed by six branches from this single axon that converge with distinct laminar distribution and range of boutons (173–648 boutons; figures 2, 3 in Rockland 2002). This organization maybe emblematic of other “columnar” patterns, where populational analysis obscurs the fact that arbors vary in laminar distribution and size.

## Functional Domains

Individual pyramidal cell clusters are frequently compared to the layout of orientation domains in visual cortex. The array is described as spanning “virtually the entire range of possible domains of the orientation map… with only a weak average bias to like-to-like coupling” (Martin et al., 2014, *n* = 33 neurons). A similar distributed mapping was found for terminal clusters referenced to cytochrome oxidase domains in macaque V1 (Yabuta and Callaway, 1998, *n* = 20 neurons). In cat area 18, superimposing 3-dimensional axonal reconstructions with optically imaged orientation domains showed the clusters as targeting iso-orientation, non-iso-orientation, and cross-orientation sites, with only about a 50% iso-orientation bias in relation to the parent neuron (Kisvarday and Eysel, 1992; and for basket cells, not discussed here, see Buzas et al., 2001; Kisvarday et al., 2002.)

Several issues are important to mention, even if briefly. First, in the visual modality, orientation preference is only one of several features for which neurons might be selective, along with, for example, ocularity, disparity, color preference, or border ownership, among others. Second, areas are not homogeneous (e.g., the cytochrome oxidase system of areas V1 and V2); and both the anatomic and functional diversity of pyramidal neuron subtypes are still under investigation (e.g., Berg et al., 2021). Third, intrinsic patches are not necessarily limited to a single system but may participate in multiple systems, either by virtue of diversity in the parent neurons or in the arrangement of synapses on postsynaptic dendrites (Ju et al., 2020). A case for multiple overlapping connectivity rules has in fact been made by simulations (Muir et al., 2011).

## Summary

Single neuron analysis of horizontal intrinsic connections clearly reveals a nuanced picture: individual neurons can have both linear (cross-columnar) and clustered terminations, and differentiated home and distal clusters. Some layer 5, corticothalamic neurons have a perisomatic termination free zone. This pattern is less suggestive of any uniform columnar or exclusively like-to-like functional organization than a means of achieving selective recombination through convergence and divergence within and across individual axons. The recombination could more generally subserve context-dependent processing, as suggested for visual cortex (Stettler et al., 2002; Martin et al., 2014, 2017; Scholl and Fitzpatrick, 2020) or, especially since the same anatomical features can be anticipated in frontal and other non-sensory areas, might figure in differential timing, neuronal synchrony, or plasticity features (i.e., more a whirligig or octopus, than a daisy). Obvious avenues for continued work include a larger sampling within and across different cortical areas, ideally in 3-dimensional tissue blocks; visualization of both the intrinsic and extrinsic arborization of multiple, identifiably tagged individual neurons; and dynamic cellular-level imaging of interconnected patches under different behavioral conditions.

## Author Contributions

The author confirms being the sole contributor of this work and has approved it for publication.

## Funding

Publication costs were generously contributed by the Department of Anatomy and Neurobiology, Boston University School of Medicine.

## Conflict of Interest

The author declares that the research was conducted in the absence of any commercial or financial relationships that could be construed as a potential conflict of interest.

## Publisher's Note

All claims expressed in this article are solely those of the authors and do not necessarily represent those of their affiliated organizations, or those of the publisher, the editors and the reviewers. Any product that may be evaluated in this article, or claim that may be made by its manufacturer, is not guaranteed or endorsed by the publisher.
